# *catena*-Poly[[bis[quinazolin-4(3*H*)-one-κ*N* ^1^]cadmium(II)]-di-μ-chlorido]

**DOI:** 10.1107/S1600536810041590

**Published:** 2010-10-23

**Authors:** Kambarali Turgunov, Ulli Englert

**Affiliations:** a S.Yunusov Institute of the Chemistry of Plant Substances, Academy of Sciences of Uzbekistan, Mirzo Ulugbek Str., 77, Tashkent 100170, Uzbekistan; b Institute of Inorganic Chemistry, RWTH Aachen, Landoltweg 1, D-52056 Aachen, Germany

## Abstract

The asymmetric unit of the title compound, [CdCl_2_(C_8_H_6_N_2_O)_2_]_*n*_, consists of one mol­ecule of the 3*H*-quinazolin-4-one ligand, one Cd^2+^ cation, which is located on a twofold axis, and one chlorido ligand in a general position. The latter bridges metal cations, forming a one-dimensional polymer along the *b* axis. The Cd⋯Cd distance along the chain is 3.7309 (7) Å. The octa­hedral coordination around the metal is completed by two ligands in a *trans* axial geometry which coordinate through the N atom in 1 position. Moderately strong classical N—H⋯O hydrogen bonds around crystallographic inversion centers cross-link adjacent polymeric chains.

## Related literature

The crystal structure of 3*H*-pyrimidin-4-one was reported by Vaillancourt *et al.* (1998 ▶). For related Cd(II) coordination polymers, see: Hu & Englert (2002 ▶); Hu *et al.* (2003 ▶); Englert & Schiffers (2006*a* ▶,*b* ▶); Cao *et al.* (2008 ▶). For a general review of halide-bridged chain polymers, see: Englert (2010 ▶). 
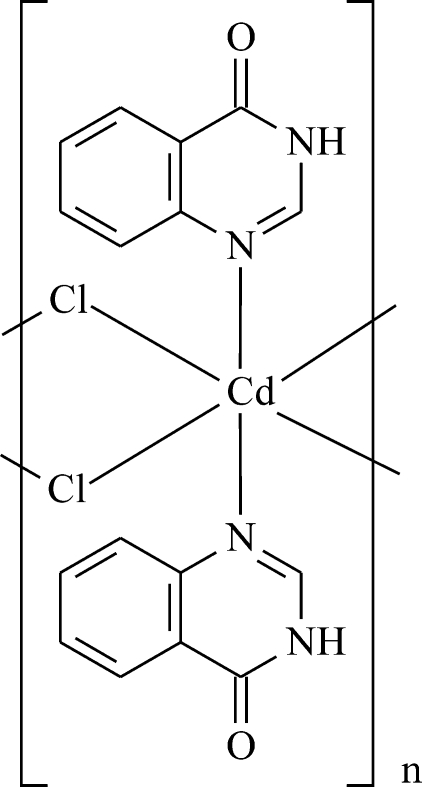


## Experimental

### 

#### Crystal data

[CdCl_2_(C_8_H_6_N_2_O)_2_]
*M* *_r_* = 475.60
Monoclinic, 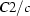

*a* = 28.839 (6) Å
*b* = 3.7309 (7) Å
*c* = 17.846 (4) Å
β = 123.26 (3)°
*V* = 1605.6 (8) Å^3^
*Z* = 4
Mo *K*α radiation
μ = 1.71 mm^−1^
*T* = 130 K
0.80 × 0.03 × 0.02 mm

#### Data collection

Bruker SMART APEX diffractometer
Absorption correction: multi-scan (*MULABS*; Blessing, 1995 ▶) *T*_min_ = 0.936, *T*_max_ = 0.958
10107 measured reflections
1983 independent reflections
1831 reflections with *I* > 2σ(*I*)
*R*_int_ = 0.081

#### Refinement

*R*[*F* ^2^ > 2σ(*F* ^2^)] = 0.043
*wR*(*F* ^2^) = 0.102
*S* = 1.16
1983 reflections
118 parameters
H atoms treated by a mixture of independent and constrained refinement
Δρ_max_ = 0.91 e Å^−3^
Δρ_min_ = −2.47 e Å^−3^

### 

Data collection: *SMART* (Bruker, 2000 ▶); cell refinement: *SAINT-Plus* (Bruker, 1999 ▶); data reduction: *SAINT-Plus*; program(s) used to solve structure: *SHELXS97* (Sheldrick, 2008 ▶); program(s) used to refine structure: *SHELXL97* (Sheldrick, 2008 ▶); molecular graphics: *XP* in *SHELXTL* (Sheldrick, 2008 ▶); software used to prepare material for publication: *SHELXL97*.

## Supplementary Material

Crystal structure: contains datablocks I, global. DOI: 10.1107/S1600536810041590/gk2309sup1.cif

Structure factors: contains datablocks I. DOI: 10.1107/S1600536810041590/gk2309Isup2.hkl

Additional supplementary materials:  crystallographic information; 3D view; checkCIF report

## Figures and Tables

**Table 1:** Hydrogen-bond geometry (Å, °)

*D*—H⋯*A*	*D*—H	H⋯*A*	*D*⋯*A*	*D*—H⋯*A*
N3—H3⋯O1^i^	0.87 (5)	1.90 (4)	2.762 (5)	172 (6)

Symmetry code: (i) 
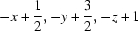
.

**Fig. 1.**
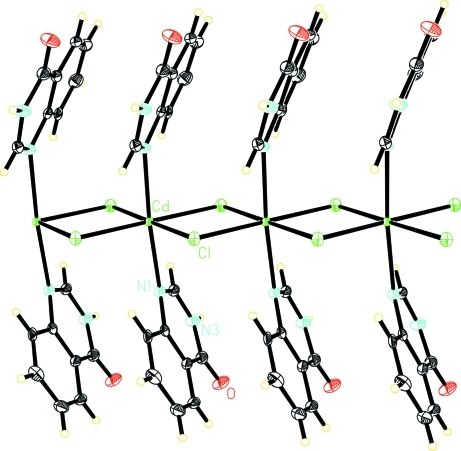
Section of the chain polymer, viewed along the c axis.

**Fig. 2.**
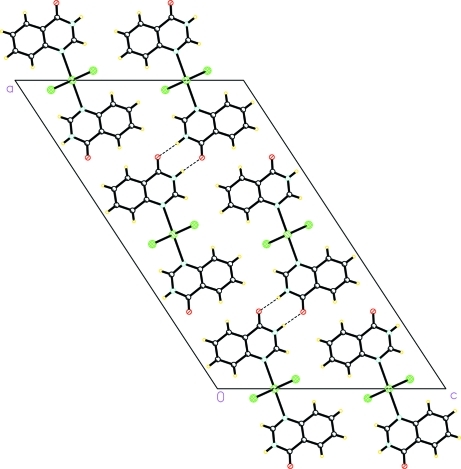
Projection of the structure along the b direction.

## supplementary crystallographic information

### Comment

The title compound represents the first crystal structure of a complex in which 3*H*-quinazolin-4-one acts as a ligand; the uncoordinated organic molecule has not been reported neither. The title compound is a chain polymer in which each Cd(II) cation is coordinated by four bridging chloro ligands in the equatorial plane and two monodentate 3*H*-quinazolin-4-one ligands in the axial positions of a pseudo-octahedron. The chain direction corresponds to the shortest lattice parameter; a section of the polymer is shown in Fig. 1. The metal···nitrogen vector and the metal–halide plane subtend an angle of 84.5 (1)°. The angle N—Cd—N^ii^ (ii:*-x, y, 1/2 - z*) amounts to 175.3 (2)°, and the dihedral angle between the least squares plane through the ligand and the metal–halide plane to 67.00 (6)°. Tilting of the ligand molecules in this structure is stabilized by intermolecular N—H···O hydrogen bonds around crystallographic inversion centers (Table 1, Fig.2).

### Experimental

A solution of 73.33 mg (0.4 mmol) of cadmium (II) chloride in 20 ml of water was added to a solution of 116.92 mg (0.8 mmol) of 3*H*-quinazolin-4-one in 30 ml of acetone. A precipitate formed immediately which was recovered by filtration. Single crystals suitable for the diffraction experiment were obtained by dissolving this precipitate in a 1:3 water:acetone mixture and slow evaporation at room temperature. The crystals grew as colourless needles.

### Refinement

Carbon-bound H atoms were positioned geometrically and treated as riding on their C atoms, with C—H distances of 0.93 Å (aromatic) and were refined with *U*_iso_(H)=1.2Ueq(C). Nitrogen-bound H atom involved in the intermolecular hydrogen bonding was located by difference Fourier synthesis and refined freely [N—H =0.87 (5) Å].

### Crystal data

**Table:**

[CdCl_2_(C_8_H_6_N_2_O)_2_]	*F*(000) = 936
*M**_r_* = 475.60	*D*_x_ = 1.967 Mg m^−^^3^
Monoclinic, *C*2/*c*	Mo *K*α radiation, λ = 0.71073 Å
Hall symbol: -C 2yc	Cell parameters from 8356 reflections
*a* = 28.839 (6) Å	θ = 2.3–28.4°
*b* = 3.7309 (7) Å	µ = 1.71 mm^−^^1^
*c* = 17.846 (4) Å	*T* = 130 K
β = 123.26 (3)°	Needle, colourless
*V* = 1605.6 (8) Å^3^	0.80 × 0.03 × 0.02 mm
*Z* = 4	

### Data collection

**Table:**

Bruker SMART APEX diffractometer	1983 independent reflections
Radiation source: fine-focus sealed tube	1831 reflections with *I* > 2σ(*I*)
graphite	*R*_int_ = 0.081
ω scans	θ_max_ = 28.4°, θ_min_ = 2.3°
Absorption correction: multi-scan (*MULABS*; Blessing, 1995)	*h* = −38→38
*T*_min_ = 0.936, *T*_max_ = 0.958	*k* = −4→4
10107 measured reflections	*l* = −23→23

### Refinement

**Table:**

Refinement on *F*^2^	Primary atom site location: structure-invariant direct methods
Least-squares matrix: full	Secondary atom site location: difference Fourier map
*R*[*F*^2^ > 2σ(*F*^2^)] = 0.043	Hydrogen site location: inferred from neighbouring sites
*wR*(*F*^2^) = 0.102	H atoms treated by a mixture of independent and constrained refinement
*S* = 1.16	*w* = 1/[σ^2^(*F*_o_^2^) + (0.045*P*)^2^] where *P* = (*F*_o_^2^ + 2*F*_c_^2^)/3
1983 reflections	(Δ/σ)_max_ < 0.001
118 parameters	Δρ_max_ = 0.91 e Å^−^^3^
0 restraints	Δρ_min_ = −2.47 e Å^−^^3^

### Special details

**Table:**

Geometry. All e.s.d.'s (except the e.s.d. in the dihedral angle between two l.s. planes) are estimated using the full covariance matrix. The cell e.s.d.'s are taken into account individually in the estimation of e.s.d.'s in distances, angles and torsion angles; correlations between e.s.d.'s in cell parameters are only used when they are defined by crystal symmetry. An approximate (isotropic) treatment of cell e.s.d.'s is used for estimating e.s.d.'s involving l.s. planes.
Refinement. Refinement of *F*^2^ against ALL reflections. The weighted *R*-factor *wR* and goodness of fit *S* are based on *F*^2^, conventional *R*-factors *R* are based on *F*, with *F* set to zero for negative *F*^2^. The threshold expression of *F*^2^ > σ(*F*^2^) is used only for calculating *R*-factors(gt) *etc*. and is not relevant to the choice of reflections for refinement. *R*-factors based on *F*^2^ are statistically about twice as large as those based on *F*, and *R*- factors based on ALL data will be even larger.

### Fractional atomic coordinates and isotropic or equivalent isotropic displacement parameters (Å^2^)

**Table:**

	*x*	*y*	*z*	*U*_iso_*/*U*_eq_	
Cd1	0.0000	0.41717 (11)	0.2500	0.02822 (14)	
Cl1	0.03110 (4)	0.9258 (3)	0.36946 (6)	0.0345 (2)	
O1	0.25065 (13)	0.8403 (9)	0.4033 (2)	0.0456 (8)	
N1	0.09451 (14)	0.4438 (9)	0.2882 (2)	0.0345 (7)	
C2	0.12966 (17)	0.4725 (11)	0.3729 (3)	0.0348 (9)	
H2	0.1180	0.4045	0.4102	0.042*	
N3	0.18220 (15)	0.5931 (11)	0.4126 (3)	0.0389 (8)	
C4	0.20342 (17)	0.7146 (12)	0.3647 (3)	0.0371 (9)	
C4A	0.16615 (17)	0.6802 (11)	0.2686 (3)	0.0341 (9)	
C5	0.18303 (17)	0.7898 (12)	0.2120 (3)	0.0373 (9)	
H5	0.2179	0.8906	0.2359	0.045*	
C6	0.14774 (18)	0.7473 (13)	0.1212 (3)	0.0410 (9)	
H6	0.1587	0.8181	0.0831	0.049*	
C7	0.09534 (19)	0.5976 (13)	0.0859 (3)	0.0406 (9)	
H7	0.0718	0.5669	0.0243	0.049*	
C8	0.07797 (18)	0.4957 (10)	0.1400 (3)	0.0343 (9)	
H8	0.0428	0.3983	0.1151	0.041*	
C8A	0.11298 (17)	0.5373 (11)	0.2328 (3)	0.0333 (8)	
H3	0.206 (2)	0.609 (13)	0.470 (3)	0.040 (13)*	

### Atomic displacement parameters (Å^2^)

**Table:**

	*U*^11^	*U*^22^	*U*^33^	*U*^12^	*U*^13^	*U*^23^
Cd1	0.0258 (2)	0.0272 (2)	0.0302 (2)	0.000	0.01445 (17)	0.000
Cl1	0.0353 (5)	0.0325 (5)	0.0343 (5)	0.0002 (4)	0.0182 (4)	0.0008 (4)
O1	0.0363 (16)	0.055 (2)	0.0443 (17)	−0.0100 (14)	0.0211 (14)	−0.0023 (15)
N1	0.0304 (16)	0.0340 (18)	0.0388 (18)	−0.0007 (14)	0.0188 (15)	−0.0008 (15)
C2	0.0326 (19)	0.033 (2)	0.040 (2)	−0.0001 (16)	0.0205 (18)	0.0015 (17)
N3	0.0307 (17)	0.047 (2)	0.0354 (19)	−0.0019 (16)	0.0158 (16)	0.0004 (17)
C4	0.033 (2)	0.035 (2)	0.041 (2)	−0.0013 (17)	0.0192 (18)	−0.0019 (18)
C4A	0.0321 (19)	0.030 (2)	0.040 (2)	−0.0001 (16)	0.0194 (18)	0.0002 (16)
C5	0.033 (2)	0.033 (2)	0.049 (2)	0.0009 (17)	0.0244 (19)	0.0025 (18)
C6	0.044 (2)	0.040 (2)	0.047 (2)	0.003 (2)	0.030 (2)	0.001 (2)
C7	0.042 (2)	0.042 (2)	0.039 (2)	0.004 (2)	0.0231 (19)	−0.0009 (19)
C8	0.034 (2)	0.0256 (19)	0.042 (2)	0.0015 (15)	0.0196 (18)	−0.0011 (15)
C8A	0.0332 (19)	0.0272 (19)	0.041 (2)	0.0031 (16)	0.0217 (18)	0.0010 (16)

### Geometric parameters (Å, °)

**Table:**

Cd1—N1	2.422 (3)	N3—H3	0.87 (5)
Cd1—N1^i^	2.422 (3)	C4—C4A	1.445 (6)
Cd1—Cl1^ii^	2.5714 (11)	C4A—C5	1.402 (6)
Cd1—Cl1^iii^	2.5714 (11)	C4A—C8A	1.403 (6)
Cd1—Cl1	2.6180 (11)	C5—C6	1.370 (6)
Cd1—Cl1^i^	2.6180 (11)	C5—H5	0.9300
Cl1—Cd1^iv^	2.5714 (11)	C6—C7	1.396 (6)
O1—C4	1.233 (5)	C6—H6	0.9300
N1—C2	1.283 (6)	C7—C8	1.363 (6)
N1—C8A	1.400 (5)	C7—H7	0.9300
C2—N3	1.350 (5)	C8—C8A	1.397 (6)
C2—H2	0.9300	C8—H8	0.9300
N3—C4	1.373 (6)		
			
N1—Cd1—N1^i^	175.31 (17)	C2—N3—H3	124 (3)
N1—Cd1—Cl1^ii^	95.13 (9)	C4—N3—H3	113 (3)
N1^i^—Cd1—Cl1^ii^	88.22 (9)	O1—C4—N3	120.7 (4)
N1—Cd1—Cl1^iii^	88.22 (9)	O1—C4—C4A	124.8 (4)
N1^i^—Cd1—Cl1^iii^	95.13 (9)	N3—C4—C4A	114.4 (4)
Cl1^ii^—Cd1—Cl1^iii^	89.06 (5)	C5—C4A—C8A	120.5 (4)
N1—Cd1—Cl1	84.90 (9)	C5—C4A—C4	120.2 (4)
N1^i^—Cd1—Cl1	91.69 (9)	C8A—C4A—C4	119.4 (4)
Cl1^ii^—Cd1—Cl1	179.01 (3)	C6—C5—C4A	119.5 (4)
Cl1^iii^—Cd1—Cl1	91.93 (4)	C6—C5—H5	120.2
N1—Cd1—Cl1^i^	91.69 (9)	C4A—C5—H5	120.2
N1^i^—Cd1—Cl1^i^	84.90 (9)	C5—C6—C7	119.8 (4)
Cl1^ii^—Cd1—Cl1^i^	91.93 (4)	C5—C6—H6	120.1
Cl1^iii^—Cd1—Cl1^i^	179.01 (3)	C7—C6—H6	120.1
Cl1—Cd1—Cl1^i^	87.08 (5)	C8—C7—C6	121.3 (4)
Cd1^iv^—Cl1—Cd1	91.93 (4)	C8—C7—H7	119.3
C2—N1—C8A	116.8 (4)	C6—C7—H7	119.3
C2—N1—Cd1	112.0 (3)	C7—C8—C8A	120.1 (4)
C8A—N1—Cd1	128.1 (3)	C7—C8—H8	120.0
N1—C2—N3	125.5 (4)	C8A—C8—H8	120.0
N1—C2—H2	117.3	C8—C8A—N1	120.1 (4)
N3—C2—H2	117.3	C8—C8A—C4A	118.8 (4)
C2—N3—C4	122.6 (4)	N1—C8A—C4A	121.1 (4)

Symmetry codes: (i) −*x*, *y*, −*z*+1/2; (ii) −*x*, *y*−1, −*z*+1/2; (iii) *x*, *y*−1, *z*; (iv) *x*, *y*+1, *z*.

### Hydrogen-bond geometry (Å, °)

**Table:**

*D*—H···*A*	*D*—H	H···*A*	*D*···*A*	*D*—H···*A*
N3—H3···O1^v^	0.87 (5)	1.90 (4)	2.762 (5)	172 (6)

Symmetry codes: (v) −*x*+1/2, −*y*+3/2, −*z*+1.
